# *VDR*, *CXCR1*, *CXCR2*, *PSCA* Polymorphisms and Recurrent Urinary Tract Infections in Women: Genetic Association Study

**DOI:** 10.1007/s00192-024-05742-2

**Published:** 2024-02-26

**Authors:** Paulomi Sarker, Glaucia Miranda Varella Pereira, Vik Khullar, Jiakun Yu, Rufus Cartwright

**Affiliations:** 1 Department of Urogynaecology, Imperial College London, London, UK; 2 Institute of Reproductive and Developmental Biology, Imperial College London, London, UK; 3 Department of Obstetrics and Gynaecology, School of Medical Sciences, University of Campinas, Campinas, Brazil; 4 University Hospitals Sussex NHS Foundation Trust, London, UK; 5 Department of Urogynaecology, Chelsea & Westminster NHS Foundation Trust, London, UK

**Keywords:** rUTI, SNPs, *VDR*, *CXCR1*, *CXCR2*, *PSCA*

## Abstract

**Introduction and hypothesis:**

Urinary tract infection (UTI) is one of the most common human infections. Evidence suggests that there might be a genetic predisposition to UTI. Previous small candidate gene studies have suggested that common variants in genes involved in the immune response to UTI could increase susceptibility to the development of recurrent UTI (rUTI). The objective was to conduct a gene association study to replicate previous gene association studies identifying single nucleotide polymorphisms (SNPs) putatively associated with rUTI in adult women.

**Methods:**

Women with a history of rUTI and healthy controls were recruited (*n* = 1,008) from gynaecology outpatient clinics. Participants completed a signed consent form and questionnaire for phenotyping. DNA was extracted from blood or saliva samples for each participant. Putative associated SNPs were identified from a comprehensive systematic review of prior gene association studies. Primers for each selected SNP were designed, and genotyping was conducted using a competitive polymerase chain reaction (PCR) method. The Chi-squared test was used to assess the association between each variant and rUTI. Genotyping quality was assessed by checking for deviation from Hardy–Weinberg equilibrium.

**Results:**

We found no association between SNPs tested in the *VDR* (*p* = 0.16, *p* = 0.09, *p* = 0.36), *CXCR1* (*p* = 0.09), *CXCR2* (*p* = 0.39), *PSCA* (*p* = 0.74) genes, and rUTI in adult women.

**Conclusions:**

To our knowledge, this is the largest study to date, finding no significant associations. Previously reported positive associations may have been due to type 1 error, or genotyping errors. Future studies should adjust for confounders and employ adequate sample sizes. A greater understanding of the genetic components associated with rUTI may influence future treatment guidelines and screening for susceptible patients.

## Introduction

Urinary tract infections (UTI) include any infection of the kidneys, ureters, bladder, and urethra [1]. The most common organism causing UTI is *Escherichia coli* [2]. Approximately 50–60% of women have experienced a UTI in their lives and 27–44% of women who have had an episode of a UTI will experience a recurrence [3]. Recurrent UTI (rUTI) is defined as more than two episodes of UTI in the last 6 months or more than three episodes in the last 12 months [2, 3]. These recurrent infections are usually caused by reinfection by the same pathogen [2].

Women with a history of rUTI also report a greater prevalence of UTI among their female relatives, suggesting a genetic predisposition to the disease [4]. A study showed a similar history of rUTI in 65.5% of mothers, 61% of daughters and 49% of sisters of 41 adult women [5]. Single nucleotide polymorphisms (SNPs) are variations in the DNA of individuals, which can have an impact on gene function and gene expression. SNPs in genes involved in the immune response to UTI could be responsible for the increased susceptibility to the development of UTI [6].

This study was aimed at replicating previous candidate gene association studies identifying SNPs putatively associated with rUTI in adult women. SNPs for analysis in this study were chosen after a systematic review and meta-analysis of all previous gene association studies for UTI in children and adults. We aimed to replicate all significantly replicated associations from those meta-analyses, selecting SNPs in the vitamin D receptor (*VDR*), *CXCR1*, *CXCR2* and *PSCA*.

Vitamin D is involved in the first line of defence against bacterial infections within the urothelium via its nuclear receptor and it has antibacterial properties [7–9]. Vitamin D promotes monocyte differentiation and inhibits lymphocyte proliferation and secretion of cytokines such as interleukin-2, interferon-γ and interleukin-12 [6]. The *VDR* gene has over 3,500 identified variants; however, rs2228570, rs1544410, rs7975232 and rs731236 are among the most widely investigated because they are both common and historically easy to genotype. Studies have linked rs1544410 *VDR* polymorphisms with increased susceptibility to rUTI; however, it has also been found that rs7975232 may in fact have a protective factor in the susceptibility of rUTI. Research indicates that rs1544410 *VDR* has an impact on how effectively vitamin D can boost the production of antimicrobial peptides. This can lead to a reduced ability to eliminate bacterial uropathogens, potentially contributing to rUTIs [7–9].

Epithelial cells have been found to form blockades to prevent the microbes from invading in response to many proinflammatory mediators such as interleukin-8 (IL-8); the most important cytokine activator of neutrophils playing [8, 9] a major role in bacterial clearance, secreted by uroepithelial cells [10, 11]. C-X-C chemokine receptor type 1 and 2 (*CXCR1* and *CXCR2*) are seven-span transmembrane receptors coupled to G proteins [10]. *CXCR1* has the highest affinity and specificity to IL-8, which is involved in the movement of neutrophils across the affected epithelial cells in a UTI [10]. In vitro studies have found that inhibition of IL-8, *CXCR1* or *CXCR2* can weaken neutrophil migration, hindering pathogen clearance [10–12]. In experimental UTI IL-8 receptor homologue-knockout mice models, the neutrophils were unable to migrate across the epithelium, leading to renal scarring and end-stage renal disease [12].

A study investigating 20 premenopausal women with a history of rUTI and 30 premenopausal women with no previous history of UTI found that *CXCR2* expression was lower in premenopausal women with rUTI [11]. They found no changes in neutrophil *CXCR1* expression between premenopausal patients with rUTI and the healthy controls. Similarly, in a study of 129 UTI patients versus healthy controls, CXCR1 (2608) GC presented a lower prevalence among UTI patients and in chronic UTI patients than in healthy controls (*p* = 0.024, *p* = 0.003). The CXCR1 (2608) C alleles also had a lower prevalence in chronic UTIs than in the controls (*p* = 0.003).

The *PSCA* gene is a glycosylphosphatidylinositol-anchored glycoprotein found on cell membranes, classified as a prostate-specific cell-surface antigen [13]. Genetic association studies investigating *PSCA* and UTI [14] found when comparing the variant rs2976388 in female and male patients there was a significantly increased risk of UTI in women [14].

As gene association studies may frequently be flawed by small sample sizes or genotyping error, we aimed to replicate prior signals, with the long-term objective of developing novel diagnostic and therapeutic targets for UTI based on these genetic insights [14]. We hypothesized that there might be a significant association between the tested SNPs and rUTI in adult women.

## Materials and Methods

### Recruitment

Following a systematic review and meta-analysis [15] of prior gene association studies, we identified previously suggested risk variants for rUTI, *VDR* rs228570, *VDR* rs731236, *VDR* rs1544410, *VDR* rs7975232, *CXCR1* rs2234671, *CXCR2* rs11574750 and *PSCA* rs297388.

We conducted a gene association study based on a sample of women who attended the gynaecology outpatient clinic at St Mary’s Hospital Paddington. Ethical approval for the overall project design was granted from the NRES Committee London-Chelsea (12/LO/0394). Participants completed a signed consent form, and then completed a 12-item International Consultation on Incontinence Questionnaire Female Lower Urinary Tract Symptoms (ICIQ-FLUTS) questionnaire [16]. This questionnaire collects information on the frequency of the 12 major lower urinary tract symptoms using a five-point ordinal scale, with the rating of the bother of each symptom on a ten-point numerical scale.

Via whole blood samples, DNA was collected from women into a 6-ml EDTA Vacutainer tube or using Oragene saliva DNA collection kits, depending on the patients’ preferences.

All participants underwent a clinical note review to gather missing data from initial recruitment. Patients who had opted in for the follow-up were contacted after 12 months with a questionnaire to complete the missing data.

### Case Definitions

We defined rUTI from self-report or review of clinical notes using the definition from NICE guidelines: “more than two episodes in the last 6 months or more than three episodes in the last 12 months” [2]. Patients who had answered “yes” under rUTI were defined as the case patients. Women who had answered “no” were defined as control patients. We excluded women who were pregnant at the time of recruitment.

### Sample Processing

Whole-blood EDTA Vacutainers and Oragene DNA Saliva tubes were stored at –20°C or divided twice to 350 µl into two Eppendorf Safe-Lock tubes and then frozen. The urine sample was sent in a universal container to the clinical microbiology laboratory to undergo microscopy and culture following standard clinical guidelines.

### DNA Extraction

The DNA was extracted using an Invitrogen iPrep robot and separated into 96-well PCR plates and stored at –20°C. The robot used a patented magnetic bead system to extract RNA or DNA. Table 1 shows the chosen SNPs with their SNP identifier and minor allele frequency.

**Table 1:** Chosen single-nucleotide polymorphism (SNPs) with their SNP identifier and minor allele frequency. The greater the minor allele frequency, the higher power the statistical analysis has

SNP	SNP identifier	Minor allele frequency
VDR	rs228570	0.23
VDR	rs731236	0.36
VDR	rs1544410	0.48
VDR	rs7975232	0.50
CXCR1	rs2234671	0.35
CXCR2	rs11574750	0.15
PSCA	rs2976388	0.50

### KASPar Genotyping

The samples were genotyped by LGC Genomics in Hertfordshire. Primer designs for each SNP were prepared using the PrimerPicker software. LGC uses a proprietary genotyping method known as KBiosciences Competitive Allele Specific PCR (KASPar). A separate 5’ sequence was designed as a forward primer for each allele. The reaction mix includes complementary oligonucleotides for these 5’ tails bound either to FAM dye or Victoria fluorescent dye with different excitation and emission wavelengths. The fluorescent dye-bound oligonucleotides are matched by two complementary oligonucleotides bound to quenchers, which reduces the fluorescence emitted. As the DNA is amplified using polymerase chain reaction, more of the fluorescent dye-labelled oligonucleotides are combined, and uncoupled from their quenchers, producing the fluorescence. Each of the two homozygotes then have only one emission wavelength, whereas the heterozygotes has fluorescence at both wavelengths. ROX, also known as carboxyrhodamine, a fluorescent reference dye, was used as a reference to normalise the samples against to account for differences in starting amounts of DNA. Each pair of primers was tested against a panel of 50 samples to check for amplification and dimorphism. Samples were run on 1,384-well plates. Genotype calls were made using KlusterCaller software. Genotyping failed for rs2228570, with no results across the samples tested. This SNP was therefore excluded from the analyses.

### Statistical Analysis

The allelic Chi-squared test was utilised to determine the relationship between vitamin D, CXCR 1/2, and *PSCA* polymorphisms and recurrent UTI, with significance set at *p* < 0.05. Deviation from Hardy–Weinberg equilibrium was calculated as a quality control to test whether the distribution of the data was consistent with expected population frequencies.

## Results

**Fig. 1:**
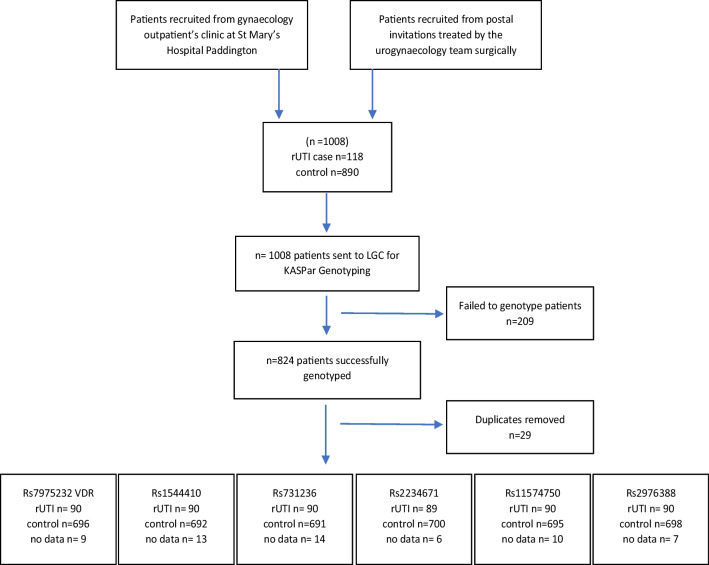
A flowchart to report the number of patients at each stage of the study

Out of 1,008 samples 979 women were recruited (rUTI *n* = 118, no rUTI *n* = 890). Figure 1 presents the number of individuals at each stage of the study. A total of 824 patients were successfully genotyped. Twenty-nine duplicates were removed and 795 participants were successfully genotyped, meeting our inclusion and exclusion criteria. These patients had data for the six SNPs tested. The number of patients out of the 795 genotyped with no data is indicated for each SNP.

The participants recruited for this gene association study were of diverse ethnicities and social backgrounds (Table 2). The analysis was not adjusted for age, BMI, and parity or menopausal status. Patients were followed up after 12 months with a postal questionnaire. Table 3 presents the gene context, SNPs, alleles and *p* values for the gene tested. We found no association between gene polymorphisms tested and recurrent urinary tract infections at a significance level of *p* < 0.05.

**Table 2:** Characteristics of the patients recruited including case and control age and body mass index (*BMI*) with standard deviation indicated in brackets

Characteristic	Average age (years)	Average BMI (kg/m^2^)
rUTI case women	59 (± 18.2)	25.8 (± 6.7)
Control women	58 (± 15.1)	25.9 (± 5.7)

*rUTI* recurrent urinary tract infection

**Table 3:** Gene context, variant single-nucleotide polymorphisms, alleles and *p* values for the genes tested in this study. *p* < 0.05 suggests statistical significance and p > 0.05 suggests no statistical significance

Gene context	Variants/SNP	Alleles	*p* value (*p* < 0.05)
VDR	rs7975232	A/C	0.16
VDR	rs1544410	C/T	0.09
VDR	rs731236	A/G	0.07
CXCR1	rs2234671	C/G	0.09
CXCR2	rs11574750	C/T	0.39
PSCA	rs2976388	A/G	0.74

Table 4 shows the frequency of each allele for the tested gene polymorphisms. Additional quality control was with Hardy–Weinberg law at a significance level of *p* < 0.05. *p* values obtained are shown on Table 4. All distributions were consistent with the Hardy–Weinberg equilibrium, suggesting no systematic genotyping error.

**Table 4:** Frequency of each allele in the case cohort compared with the control cohort

	Frequency	Frequency	Hardy–Weinberg *p* value (*p* < 0.05)
rs7975232 VDR			
Case *n* = 90	Frequency of A: 226.00	Frequency of C: 73.00	0.85
Control *n* = 696	Frequency of A: 745.00	Frequency of C: 651.00	
rs1544410 VDR			
Case *n* = 90	Frequency of C: 98.00	Frequency of T: 80.00	0.99
Control *n* = 692	Frequency of C: 853.00	Frequency of T: 532.00	
rs731236 VDR			
Case *n* = 90	Frequency of A: 99.00	Frequency of G: 79.00	0.96
Control *n* = 691	Frequency of A: 865.00	Frequency of G: 515.00	
rs2234671 CXCR1			
Case *n* = 90	Frequency of C: 171.00	Frequency of G: 7.00	0.06
Control *n* = 695	Frequency of C: 1298.00	Frequency of G: 102.00	
rs11574750 CXCR2			
Case *n* = 89	Frequency of C: 170.00	Frequency of T: 10.00	0.43
Control *n* = 700	Frequency of C: 1334.00	Frequency of T: 58.00	
rs2976388 PSCA			
Case *n* = 90	Frequency of A: 72.00	Frequency of G: 104.00	0.25
Control *n* = 698	Frequency of A: 591.00	Frequency of G: 809.00	

We ran post hoc stratified analyses, testing the impact of each variant for pre-menopausal and post-menopausal women. Among the 12 additional tests we found a possible association (marked with *) for the rs11574750 variant of *CXCR2* in post-menopausal women only (*p* = 0.02; Table 5).

**Table 5:** Gene context, variant single-nucleotide polymorphisms, alleles and *p* values for the genes tested in pre-menopausal (age ≤ 51) women and post-menopausal (age ≥ 52).

Gene context	Variants/SNP	Alleles	Pre-menopausal *p* value (*p* < 0.05)	Post-menopausal *p* value
VDR	rs7975232	A/C	0.09	0.47
VDR	rs1544410	C/T	0.15	0.62
VDR	rs731236	A/G	0.16	0.56
CXCR1	rs2234671	C/G	0.18	0.45
CXCR2	rs11574750	C/T	0.25	0.02*
PSCA	rs2976388	A/G	0.16	0.81

*p < 0.05 suggests statistical significance

## Discussion

Our study found no association between any of the tested SNPs and the susceptibility to rUTI. No genotyping error was detected, with the distribution of alleles all consistent with Hardy–Weinberg equilibrium.

*CXCR1* and *CXCR2* are specific receptors for IL-8 that recruit neutrophils for bacterial clearance through a chemotactic gradient created by the infected urothelium, activating the relevant chemokine [17]. The functional state of these recruited neutrophils is determined by the expression of chemokine receptors in the urinary tract [18]. Furthermore, vitamin D predominantly plays a role in the immunological and inflammatory process by enhancing phagocytosis via macrophage activation [9]. Both the innate and adaptive immune systems require vitamin D for improved response and functioning during infection and inflammation owing to its role in white blood cell activation, interleukin and cytokine stimulation signalling [8].

Limited research has conducted on the association between *VDR* polymorphisms in adult women and rUTI. Earlier studies have supported an association among polymorphisms in the *VDR* gene, UTI recurrence [7] and children [9]. Our study participants were adult women, and the findings may not be generalizable to children or men.

*CXCR1* and *CXCR2* are specific receptors for IL-8, which plays a role in recruiting neutrophils and bacterial clearance [19]. Rs2234671 causes the amino acid serine to replace threonine at amino acid residue 276 of the CXCR1 protein [19]. *CXCR1* is also linked to urinary IL-8 concentrations, implying that rs2234671 might be involved in the regulation of neutrophil recruitment towards the infected section of the urinary tract in response to IL-8 generated by the uroepithelial cells [19]. This mutation is suggested to have functional significance for the binding affinity of IL-8 to the *CXCR1* receptor [19]. Despite these findings, the first meta-analysis conducted between *CXCR1* and *CXCR2* polymorphisms and susceptibility to UTI showed no correlation between the SNP rs2234671 and susceptibility for UTI in adults [19]. However, this review did suggest an increased risk of developing UTI and *CXCR1* rs223467 in children [19].

There is limited research around the association with variants in *PSCA* and urinary tract infections. One earlier genome wide association study (GWAS) found an association between UTIs and variants in PSCA rs2976388 [14]. Despite adequate power for such a large effect, we were unable to replicate this association, suggesting that the earlier findings could have been due to type 1 error, which is common in GWAS.

### Relevance for Clinical Practice

Identifying women at risk of rUTI may help us to target prophylactic measures [20]. Further clinical studies are required to determine optimal prophylaxis regimes for women at risk of rUTI [21, 22]. Novel strategies in the management of recurrent UTI might be found by identifying the underlying pathophysiology of those susceptible to rUTI [22]. Identifying causal genetic pathways may also allow manipulation of host defence mechanisms, providing new options for treating UTI [23].

### Strengths and Limitations

The case definition of self-reported rUTI may have introduced misclassification bias. This can have unpredictable effects on the outcome of our analyses and could have an impact on the observed non-significant results across all variants. Further studies would require accurate diagnosis of UTI and removal of the self-reported element to prevent misclassification bias. Not all UTIs present with typical symptoms, and some can be asymptomatic [24]. Better discriminatory power might have been obtained if we had separated our sample into pre- and post-menopausal women, who might have different causal mechanisms. Although the SNPs were all moderately common, SNP rs11574750 in *CXCR2* had a MAF of 0.15, which limits the power of the analysis. Overall, for smaller effect sizes, insufficient power may have contributed to our results differing from those of previous studies.

Bias in genotyping is less likely. The phenotypes were collected independently from the DNA samples; hence, differential bias is not likely. Extensive quality control was applied at every stage of preparation of the genotype data. Variability in the quality comparing DNA prepared from blood or saliva resulted in exclusion of samples prepared from saliva. Differential misclassification is not a risk owing to the fact that samples were processed and analysed blind.

There was no deviation from the Hardy–Weinberg equilibrium, suggesting no major genotyping errors. Out of the 29 duplicates three discrepancies (10%) in the alleles were received. In these cases, the blood samples were deemed more accurate over the saliva samples. This suggests that there might have been discrepancies throughout saliva samples that did not have blood duplicates. Although Hardy–Weinberg equilibrium was assessed, bias towards the null is still possible from any undetected genotyping errors.

## Conclusions

In conclusion, genetic susceptibility to recurrent UTI remains poorly understood. Multiple factors, such as gene–gene and gene–environmental interactions could be involved [20]. Research has indicated that vitamin D, *CXCR1* and *CXCR2* have roles in the signalling of interleukins and bacterial clearance during a UTI. Despite this, our study shows no specific association between the genetic predictors *VDR*, *CXCR1*, *CXCR2* and *PSCA* and rUTI. To have a greater understanding of the pathogenesis of UTI is a crucial step in establishing therapeutic strategies and preventive measures for this disease.

## Data Availability

The data analysed by the present study may be made available by the corresponding author upon reasonable request.

## References

[1] NICE guidelines. Urinary tract infection: antimicrobial prescribing. NICE. 2018.

[2] Al-Badr A, Al-Shaikh G (2013). Recurrent urinary tract infections management in women: a review. Sultan Qaboos Univ Med J.

[3] NICE guidelines. Urinary tract infection: antimicrobial prescribing. NICE. 2018 delete??.

[4] Stauffer CM, van der Weg B, Donadini R, Ramelli GP, Marchand S, Bianchetti MG (2004). Family history and behavioral abnormalities in girls with recurrent urinary tract infections: a controlled study. J Urol.

[5] Hopkins WJ, Uehling DT, Wargowski DS (1999). Evaluation of a familial predisposition to recurrent urinary tract infections in women. Am J Med Genet.

[6] Aslan S, Akil I, Aslan G, Onay H, Ozyurt BC, Ozkinay F (2012). Vitamin D receptor gene polymorphism in children with urinary tract infection. Pediatr Nephrol.

[7] Becerra-Loaiza DS, Sánchez-Zazueta JG, Ochoa-Ramírez LA, Velarde-Rodríguez I, Rodríguez-Millán J, Velarde-Félix JS (2018). Study on vitamin D receptor gene polymorphisms in patients with urinary tract infections conducted in Northwestern Mexico. Rev Mex Urol.

[8] Ali SB, Perdawood D, Abdulrahman R, Al Farraj DA, Alkubaisi NA (2020). Vitamin D deficiency as a risk factor for urinary tract infection in women at reproductive age. Saudi J Biol Sci.

[9] Mahyar A, Ayazi P, Safari S, Dalirani R, Javadi A, Esmaeily S (2018). Association between vitamin D and urinary tract infection in children. Korean J Pediatr.

[10] Frendéus B, Godaly G, Hang L, Karpman D, Lundstedt AC, Svanborg C (2000). Interleukin 8 receptor deficiency confers susceptibility to acute experimental pyelonephritis and may have a human counterpart. J Exp Med.

[11] Smithson A, Sarrias MR, Barcelo J (2005). Expression of interleukin-8 receptors (CXCR1 and CXCR2) in premenopausal women with recurrent urinary tract infections. Clin Vaccine Immunol.

[12] Olszyna DP, Florquin S, Sewnath M (2001). CXC chemokine receptor 2 contributes to host defense in murine urinary tract infection. J Infect Dis.

[13] Xu L-P, Qiu H-B, Yuan S-Q, Chen Y-M, Zhou Z-W, Chen Y-B (2020). Downregulation of PSCA promotes gastric cancer proliferation and is related to poor prognosis. J Cancer.

[14] Tian C, Hromatka BS, Kiefer AK (2017). Genome-wide association and HLA region fine-mapping studies identify susceptibility loci for multiple common infections. Nat Commun.

[15] Yu J, Miranda VPG, Allen BK (2023). Genetic polymorphisms associated with UTI in children and adults: a systematic review and meta-analysis. Am J Obstet Gynecol.

[16] Brookes ST, Donovan JL, Wright M, Jackson S, Abrams P (2004). A scored form of the Bristol Female Lower Urinary Tract Symptoms questionnaire: data from a randomized controlled trial of surgery for women with stress incontinence. Am J Obstet Gynecol.

[17] Godaly G, Ambite I, Puthia M (2016). Urinary tract infection molecular mechanisms and clinical translation. Pathogens.

[18] Mak RH, Kuo HJ (2006). Pathogenesis of urinary tract infection: an update. Curr Opin Pediatr.

[19] Han S, Lu Y, Chen M, Xu Y, Wang Y (2019). Association between interleukin 8-receptor gene (CXCR1 and CXCR2) polymorphisms and urinary tract infection: evidence from 4097 subjects. Nephrology.

[20] Zaffanello M, Malerba G, Cataldi L (2010). Genetic risk for recurrent urinary tract infections in humans: a systematic review. J Biomed Biotechnol.

[21] Scholes D, Hooton TM, Roberts PL, Gupta K, Stapleton AE, Stamm WE (2005). Risk factors associated with acute pyelonephritis in healthy women. Ann Intern Med.

[22] Karoly E, Fekete A, Banki NF (2007). Heat shock protein 72 (HSPA1B) gene polymorphism and Toll-like receptor (TLR) 4 mutation are associated with increased risk of urinary tract infection in children. Pediatr Res.

[23] Scherberich JE, Hartinger A (2008). Impact of Toll-like receptor signalling on urinary tract infection. Int J Antimicrob Agents.

[24] Bono MJ, Reygaert WC. Urinary tract infection. In: StatPearls. StatPearls Publishing; 2021.

